# Older women with non-ST-elevation acute coronary syndrome undergoing invasive or conservative management: an individual patient data meta-analysis

**DOI:** 10.1093/ehjopen/oeae093

**Published:** 2024-10-26

**Authors:** Francesca Rubino, Graziella Pompei, Gregory B Mills, Christos P Kotanidis, Claudio Laudani, Bjørn Bendz, Erlend S Berg, David Hildick-Smith, Geir Hirlekar, Nuccia Morici, Aung Myat, Nicolai Tegn, Juan Sanchis Forés, Stefano Savonitto, Stefano De Servi, Vijay Kunadian

**Affiliations:** Translational and Clinical Research Institute, Faculty of Medical Sciences, Newcastle University, 4th Floor William Leech Building, Newcastle upon Tyne NE2 4HH, UK; Department of Cardiology, HartCentrum, Ziekenhuis aan de Stroom (ZAS) Middelheim, Lindendreef 1, 2020 Antwerp, Belgium; Translational and Clinical Research Institute, Faculty of Medical Sciences, Newcastle University, 4th Floor William Leech Building, Newcastle upon Tyne NE2 4HH, UK; Cardiovascular Institute, Azienda Ospedaliero-Universitaria di Ferrara, Via Aldo Moro 8, 44124 Cona, FE, Italy; Translational and Clinical Research Institute, Faculty of Medical Sciences, Newcastle University, 4th Floor William Leech Building, Newcastle upon Tyne NE2 4HH, UK; Cardiothoracic Centre, Freeman Hospital, Newcastle upon Tyne Hospitals NHS Foundation Trust, Freeman Rd, High Heaton, Newcastle upon Tyne, NE7 7DN, UK; Translational and Clinical Research Institute, Faculty of Medical Sciences, Newcastle University, 4th Floor William Leech Building, Newcastle upon Tyne NE2 4HH, UK; Cardiothoracic Centre, Freeman Hospital, Newcastle upon Tyne Hospitals NHS Foundation Trust, Freeman Rd, High Heaton, Newcastle upon Tyne, NE7 7DN, UK; Division of Cardiology, Azienda Ospedaliero Universitaria Policlinico ‘G. Rodolico-San Marco’, University of Catania, Via S. Sofia 78, 95123 Catania, Italy; Department of Cardiology, Oslo University Hospital, Postboks 4950 Nydalen, OUS HF Rikshospitalet, 0424 Oslo, Norway; Division of Medicine, Institute of Clinical Medicine, University of Oslo, P.O box 1171 Blindern, 0318 Oslo, Norway; Department of Cardiology, Oslo University Hospital, Postboks 4950 Nydalen, OUS HF Rikshospitalet, 0424 Oslo, Norway; Division of Medicine, Institute of Clinical Medicine, University of Oslo, P.O box 1171 Blindern, 0318 Oslo, Norway; Sussex Cardiac Centre, University Hospitals Sussex NHS Foundation Trust, Lyndhurst Road, Worthing, BN11 2DH, West Sussex, UK; Department of Molecular and Clinical Medicine, Institute of Medicine, Gothenburg University, Gothenburg, SU Sahlgrenska, 41345 Gothenburg, Sweden; Department of Cardiology, Sahlgrenska University Hospital, Blå Stråket 5, 413 45 Gothenburg, Sweden; Department of Cardiac Rehabilitation, IRCCS Fondazione Don Carlo Gnocchi, Via Carlo Girola 30, 20162 Milan, Italy; Department of Cardiology, Medpace, Vitners' Place 68, Upper Thames Street, London EC4V 3BJ, UK; Department of Cardiology, Oslo University Hospital, Postboks 4950 Nydalen, OUS HF Rikshospitalet, 0424 Oslo, Norway; Division of Medicine, Institute of Clinical Medicine, University of Oslo, P.O box 1171 Blindern, 0318 Oslo, Norway; Department of Cardiology, Hospital Clinico Universitario, INCLIVA, Universitat de Valencia, CIBER-CV, Avda Blasco Ibáñez 17, 46010, Valencia, Spain; Department of Cardiology, Clinica San Martino, Via Paradiso angolo Via Selvetta, 23864, Malgrate, Italy; Department of Molecular Medicine, University of Pavia, Viale Golgi 19, 27100 Pavia, Italy; Translational and Clinical Research Institute, Faculty of Medical Sciences, Newcastle University, 4th Floor William Leech Building, Newcastle upon Tyne NE2 4HH, UK; Cardiothoracic Centre, Freeman Hospital, Newcastle upon Tyne Hospitals NHS Foundation Trust, Freeman Rd, High Heaton, Newcastle upon Tyne, NE7 7DN, UK

**Keywords:** Conservative management, Non-ST-elevation acute coronary syndrome, Older women, Routine invasive approach

## Abstract

**Aims:**

Women and older patients are underrepresented in randomized controlled trials (RCTs) investigating treatment strategies following acute coronary syndrome. This study aims to evaluate the benefit of invasive vs. conservative strategy of older women with non-ST-elevation acute coronary syndrome (NSTEACS).

**Methods and results:**

This analysis from an individual patient data meta-analysis included six RCTs comparing an invasive management with a conservative management in older NSTEACS patients. The primary endpoint was the composite of all-cause mortality or myocardial infarction (MI). Secondary endpoints included all-cause mortality, cardiovascular death, MI, urgent revascularization, and stroke. Follow-up time was censored at 1 year. In total, 717 women [median age 84.0 (interquartile range 81.0–87.0) years] were included. The primary endpoint occurred in 21.0% in the invasive strategy vs. 27.8% in the conservative strategy [hazard ratio (HR) 0.77, 95% confidence interval (CI) 0.52–1.13, *P* = 0.160 using random effect] at 1-year follow-up. The invasive management was associated with reduced risk of MI (HR 0.49, 95% CI 0.32–0.73, *P* < 0.001) and urgent revascularization (HR 0.44, 95% CI 0.20–0.98, *P* = 0.045). No significant differences were identified in the risk of all-cause mortality, cardiovascular death, and stroke. Among males, there was no significant association between the treatment strategy and primary or secondary endpoints.

**Conclusion:**

An invasive strategy compared with a conservative strategy did not reduce the composite outcome of all-cause mortality or MI in older NSTEACS women at 1-year follow-up. An invasive strategy reduced the individual risk of MI and urgent revascularization. Our results support the beneficial role of the invasive strategy in older NSTEACS women.

**Registration:**

This meta-analysis is registered with PROSPERO (CRD42023379819).

## Graphical abstract

**Graphical Abstract:**
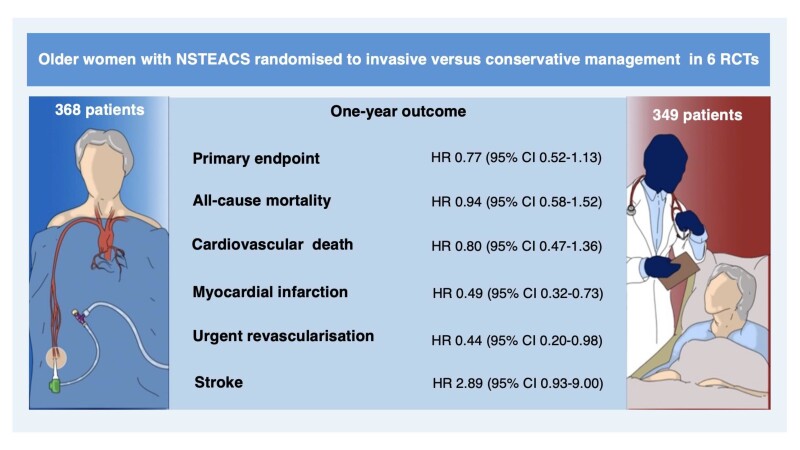

## Introduction

Ischaemic heart disease is the leading cause of mortality in women worldwide and its incidence increases with age.^1^ Among younger patients with acute coronary syndromes (ACS), women represent a minority and have less extensive coronary disease compared with men, likely due to the protective effect of oestrogen. However, the proportion of women with ACS equalizes that of men among patients older than 80 years.^2^ Although the awareness of cardiovascular disease in women and sex-specific risk factors has increased over the past years, women with cardiovascular disease, especially older patients, are still under-researched, under-diagnosed, and under-treated.^1,3^ International guidelines for the management of ACS have highlighted the under-representation of women and older patients in large randomized controlled trials (RCTs).^4–6^ There are sex-related differences in the clinical presentation of patients with ischaemic heart disease.^7^ Older women with ACS present with higher burden of frailty, comorbidities, and prevalence of cardiovascular risk factors than men, although with a lower burden of prior cardiovascular events due to delayed development of coronary disease.^8,9^

Routine early or selective invasive coronary angiography and subsequent coronary revascularization, if feasible, is recommended (class of recommendation I, level of evidence A) in patients with high-risk non-ST-elevation ACS (NSTEACS).^4^ However, women are less likely to receive invasive investigation, timely revascularization, and guideline-recommended medical therapy compared with men.^10,11^ In addition, data about clinical outcomes and invasive management in older women with NSTEACS are inconsistent and poorly understood.^12–16^

This study aims to evaluate the benefit of an invasive strategy vs. conservative strategy on clinical outcomes in older women with NSTEACS using individual patient data (IPD) from six dedicated RCTs.^17–23^

## Methods

### Study design

This study is an analysis of an IPD meta-analysis involving six RCTs comparing an invasive strategy vs. a conservative strategy in older patients with NSTEACS, with a specific focus on female patients (CRD42023379819).^17–23^ Analysis on male patients were included in the Supplementary material to provide additional information. The search algorithm has been published in the original meta-analysis and conducted according to the PRISMA-IPD guidance.^17^ The key features of the six RCTs are provided in the Supplementary material (see Supplementary material online, *Table S1*).^17^

### Comparison groups and clinical endpoints

Women and men aged ≥70 years with a diagnosis of NSTEACS were included in the study.

Non-ST-elevation ACS was defined as non-ST-elevation myocardial infarction (MI) or unstable angina with or without electrocardiographic changes and troponin rise according to the European guidelines.^4^

All the variables collected were standardized by aligning the definitions of clinical conditions and measurements. A unified data set was created from the different sources, and the accuracy of the data was confirmed by replicating the published results from all included trials and was validated by two independent reviewers (C.P.K. and G.B.M.).^17^

Early invasive management involved coronary angiography within 72 h and coronary revascularization by either percutaneous coronary intervention (PCI) or coronary artery bypass graft (CABG), if indicated, alongside optimal medical therapy. Initially conservative management comprised guideline-directed medical therapy, with coronary angiography only for urgent clinical indications including refractory ischaemia, recurrent MI, heart failure of ischaemic origin, or malignant ventricular arrhythmias.

The primary endpoint was a composite of all-cause mortality or MI. All-cause mortality, cardiovascular death, MI, urgent revascularization, and stroke were included as secondary endpoints as originally defined by the included trials (see Supplementary material online, *Table S2*). All endpoints had been centrally adjudicated in the individual trials. Subgroup analyses of the female population were performed according to age (70–80, 81–89, and ≥90 years), diabetes mellitus, previous MI, PCI, CABG, and troponin positivity to explore the risk of primary endpoint and MI.

### Statistical analysis

Categorical variables are reported as counts and percentages, and *P*-values for these comparisons were obtained using *χ*^2^ or Fisher’s exact test, as appropriate. Distribution of continuous variables was assessed using the Shapiro–Wilk test and reported as mean and standard deviation for normally distributed variables or median and interquartile range (IQR) for non-normally distributed variables, and *P*-values were obtained using Student’s *t*-test or Mann–Whitney *U* test as appropriate.

Outcomes were assessed as time-to-first event and according to the intention-to-treat principle. Cumulative incidences were computed according to the Kaplan–Meier method, and *P*-values were derived using log-rank test. For each outcome, comparisons were obtained using one-stage mixed model meta-analytic method with a random-study effect. The analyses were performed using Cox proportional hazard regression model with a random effects factor to account for variation in treatment effect across studies and with a fixed effect. Sensitivity analysis was conducted using multivariable adjustment based on random effect and fixed effect Cox model accounting for age, hypertension, and diabetes mellitus as cardiovascular risk factors. All results were reported as hazard ratio (HR) and 95% confidence interval (CI) with *P*-values. The interaction *P*-values were calculated by adding an interaction term between treatment and the tested factor variable in the Cox model. The follow-up time was censored at 1-year post-enrolment for all outcomes across the studies. For all statistical tests, alpha was set at 0.05. All analyses were conducted with R version 4.3.1 and R Studio version 1.4.1106 (R Foundation for Statistical Computing, Vienna, Austria).

## Results

### Baseline characteristics

A total of 717 women and 762 men with a median age of 84.0 years (IQR 81.0–87.0) were analysed (*Table 1* and Supplementary material online, *Table S3*). Women had fewer previous history of MI, PCI, or CABG than men (see Supplementary material online, *Table S3*). Comorbidities and medical therapy at discharge were similar between invasive and conservative groups in the female population (*Table 1*).

**Table 1:** Baseline characteristics according to the management strategy

Variables	Overall women population	Invasive management	Conservative management	*P*-value
	*n* = 717	*n* = 368	*n* = 349	
**Demographic data**				
Age, years (IQR)	84.0 (81.0–87.0)	84.0 (81.0–87.0)	84.0 (81.0–87.0)	0.677
**Past medical history**				
Hypertension, *n* (%)	527 (73.5)	275 (74.7)	252 (72.29)	0.421
Missing data, (%)	6 (0.8)	4 (1.1)	2 (0.6)	
Diabetes mellitus, *n* (%)	211 (29.4)	117 (31.8)	94 (26.9)	0.157
Missing data, (%)	5 (0.7)	4 (1.1)	1 (0.3)	
Smoking status				0.679
Active smokers, (%)	33 (4.6)	17 (4.6)	16 (4.6)	
Former smokers, (%)	127 (17.7)	59 (16.0)	68 (19.5)	
Never smokers, (%)	383(53.6)	202 (54.9)	181 (51.9)	
Missing data, (%)	174 (24.3)	90 (24.5)	84 (24.07)	
Previous MI, (%)	200 (27.9)	108 (29.3)	92 (26.4)	0.369
Missing data, (%)	8 (1.1)	6 (1.6)	2 (0.6)	
Previous CABG, (%)	54 (7.5)	26 (7.1)	28 (9.0)	0.737
Missing data, (%)	7 (1.0)	4 (1.1)	3 (0.9)	
Previous PCI, (%)	98 (13.7)	53 (14.6)	45 (12.9)	0.592
Missing data, (%)	8 (1.1)	6 (1.6)	2 (0.6)	
Previous stroke, *n* (%)	106 (14.8)	63 (17.7)	43 (12.3)	0.083
Missing data, (%)	6 (0.8)	4 (1.1)	2 (0.6)	
Killip class				0.633
1	461 (64.3)	234 (63.6)	227 (65.0)	
2	121 (16.9)	58 (15.8)	63 (18.1)	
3	12 (1.7)	8 (2.2)	4 (1.1)	
4	2 (0.3)	1 (0.3)	1 (0.3)	
Missing data, (%)	121 (16.9)	67 (18.2)	54 (15.5)	
**Medical therapy at discharge**				
Aspirin, *n* (%)	587 (81.9)	296 (80.4)	291 (83.4)	0.555
Missing data, (%)	17 (2.4)	11 (3.0)	6 (1.7)	
P2Y12 inhibitors, (%)	582 (81.2)	290 (78.8)	292 (83.7)	0.206
Missing data, (%)	15 (2.1)	10 (2.7)	5 (1.4)	
Beta-blockers, *n* (%)	451 (62.9)	225 (61.1)	226 (64.8)	0.510
Missing data, (%)	16 (2.2)	11 (3.0)	5 (1.4)	
ACE inhibitors, (%)	328 (45.7)	174 (47.3)	154 (44.1)	0.346
Missing data, (%)	17 (2.4)	11 (3.0)	6 (1.7)	
Anticoagulation, (%)	86 (12.0)	43 (11.7)	43 (12.3)	0.864
Missing data, (%)	58 (8.1)	29 (7.9)	29 (8.3)	
Statin, *n* (%)	564 (78.7)	292 (79.3)	272 (77.9)	0.461
Missing data, (%)	17 (2.4)	11 (3.0)	6 (1.7)	

ACE, angiotensin converting enzyme inhibitors; CABG, coronary artery bypass graft; IQR, interquartile range; MI, myocardial infarction; *n*, number; PCI, percutaneous coronary artery disease.

### Primary and secondary endpoints

**Figure 1:**
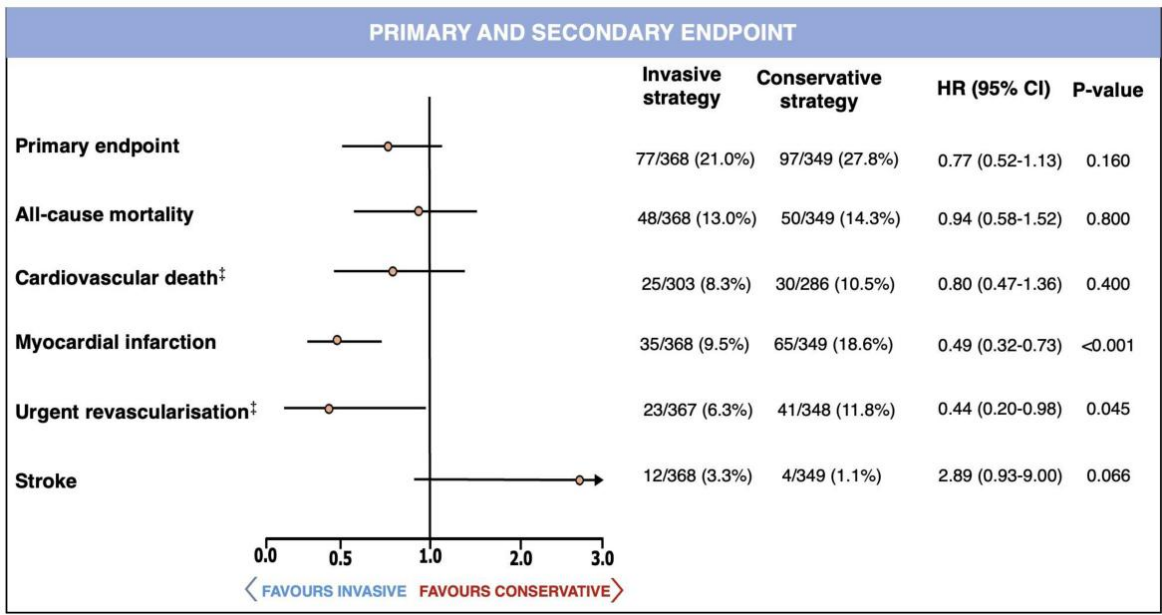
Unadjusted Cox regression analysis for primary and secondary endpoints at 1-year follow-up. CI, confidence interval; HR, hazard ratio. ^‡^For those variables with missing outcomes data, values are reported as *n*/available observations (%).

The primary endpoint occurred in 24.3% of the study population with numerically fewer events in women randomized to an invasive strategy vs. a conservative strategy (21.0% vs. 27.8%) (*Figure 1* and Supplementary material online, *Figure S1*). The unadjusted Cox regression model using random effect did not show significant association between the composite primary endpoint and the treatment strategy (unadjusted HR 0.77, 95% CI 0.52–1.13, *P* = 0.160) (*Figure 1*).

However, the adjusted Cox regression model using random effect revealed a significantly reduced risk of primary endpoint in participants allocated to invasive strategy (adjusted HR 0.71, 95% CI 0.53–0.96, *P* = 0.028) (see Supplementary material online, *Figure S2*). Using the fixed effect approach, the association between invasive strategy and primary endpoint was statistically significant in the unadjusted and adjusted analysis (see Supplementary material online, *Table S4*).

**Figure 2:**
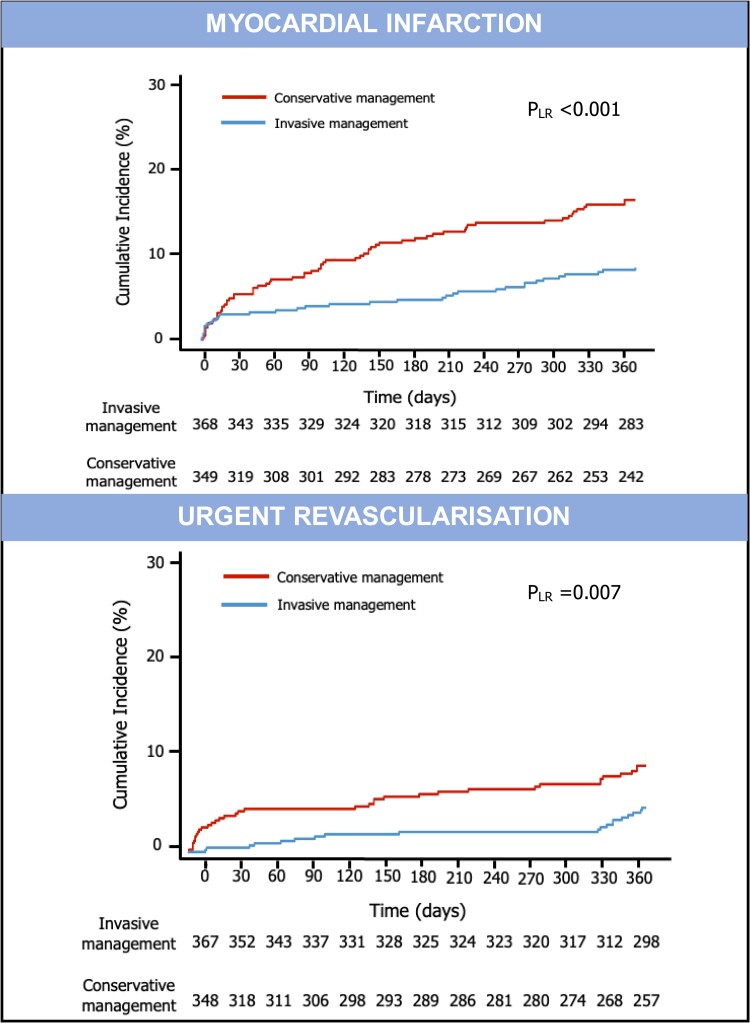
Myocardial infarction and urgent revascularization incidence in older women undergoing invasive or conservative management. Kaplan–Meier curves show higher cumulative incidence of myocardial infarction or urgent revascularization in older women undergoing conservative management than in those undergoing invasive treatment. LR, log-rank.

Women allocated to an invasive strategy had lower incidence and risk of MI than women allocated to conservative strategy (9.5% vs. 18.6%) with an unadjusted HR of 0.49 (95% CI 0.32–0.73, *P* < 0.001) and adjusted HR of 0.44 (95% CI 0.20–0.98, *P* < 0.001) using random effect (*Figures 1* and *2*; Supplementary material online, *Figure S2*). Women allocated to an invasive strategy vs. conservative strategy had lower incidence and risk of urgent revascularization (6.3% vs. 11.8%; unadjusted HR 0.44, 95% CI 0.20–0.98, *P* = 0.045 and adjusted HR 0.43, 95% CI 0.20–0.93, *P* = 0.032 using random effect) (*Figures 1* and *2*; Supplementary material online, *Figure S2*).

Allocation to invasive strategy was not significantly associated with all-cause mortality (unadjusted HR 0.94, 95% CI 0.58–1.52, *P* = 0.800 and adjusted HR 0.90, 95% CI 0.60–1.33, *P* = 0.590 using random effect) or cardiovascular death (unadjusted HR 0.80, 95% CI 0.47–1.36, *P* = 0.400 and adjusted HR 0.78, 95% CI 0.46–1.33, *P* = 0.360 using random effect) (*Figure 1*; Supplementary material online, *Figures S2* and *S3*).

The incidence of stroke tended to be higher in the invasive strategy group (3.3% vs. 1.1%) with unadjusted HR 2.89 (95% CI 0.93–9.00, *P* = 0.066) and adjusted HR 2.58 (95% CI 0.94–9.15, *P* = 0.062) using random effect (*Figure 1*; Supplementary material online, *Figures S2* and *S4*). The unadjusted and adjusted HR by applying the fixed effect model confirmed the results of the Cox regression analysis using the random effect approach for all secondary endpoints (see Supplementary material online, *Table S4*). The unadjusted and adjusted HR by using the random effect model did not demonstrate any significant association between the treatment strategy and the primary or secondary endpoints among men (see Supplementary material online, *Table S5*).

### Subgroup analysis

**Figure 3:**
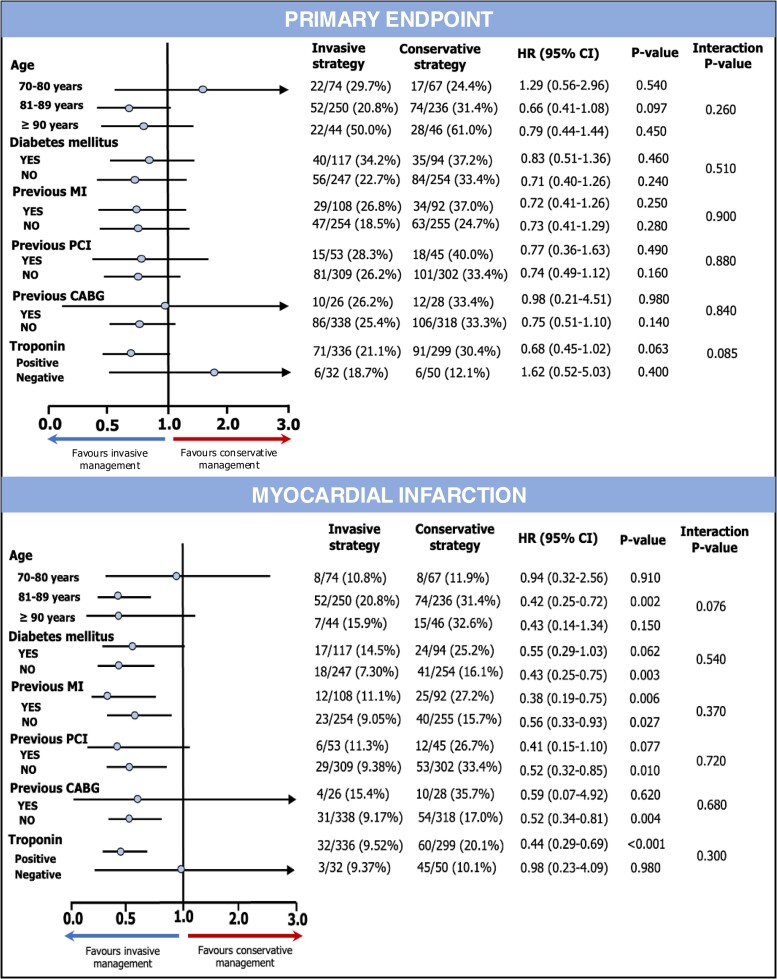
Primary endpoint and myocardial infarction in subgroup analysis. Forest plots show unadjusted Cox regression analysis using random effect in older women aged 70–80 years, 81–89 years, and ≥90 years, with or without diabetes mellitus, previous history of myocardial infarction, previous revascularization with percutaneous coronary intervention, or coronary artery bypass graft and positive or negative troponin. CI, confidence interval; CABG, coronary artery bypass graft; HR, hazard ratio; MI, myocardial infarction; PCI, percutaneous coronary intervention.

The test for interaction did not demonstrate significant association between primary endpoint or MI and any of the analysed subgroups stratified according to age, diabetes mellitus, prior PCI, prior CABG, and positive troponin (*Figure 3*). The invasive strategy was not significantly associated with primary endpoint in any of the subgroups (*Figure 3*). On the other hand, the invasive strategy demonstrated to reduce the risk of MI in women aged 81–89 years (unadjusted HR 0.42, 95% CI 0.25–0.72, *P* = 0.002), without previous history of diabetes mellitus (unadjusted HR 0.43, 95% CI 0.25–0.75, *P* = 0.003), with or without previous MI (respectively, unadjusted HR 0.38, 95% CI 0.19–0.75, *P* = 0.006; unadjusted HR 0.56, 95% CI 0.33–0.93, *P* = 0.027), previous PCI (unadjusted HR 0.52, 95% CI 0.32–0.85, *P* = 0.010) or CABG (unadjusted HR 0.52, 95% CI 0.34–0.81, *P* = 0.004), and positive troponin (unadjusted HR 0.44, 95% CI 0.29–0.69, *P* < 0.001) (*Figure 3*).

## Discussion

The present analysis using IPD demonstrates that in older women with NSTEACS, an invasive strategy in an unadjusted analysis did not reduce the risk of the primary composite endpoint of all-cause mortality or MI at 1-year follow-up, in comparison with a conservative strategy. However, the risk of MI and urgent revascularization are lower in older women with NSTEACS allocated to invasive strategy than those allocated to conservative strategy. The positive effect of an invasive strategy on MI seems to be significantly greater in women with elevated troponin levels, without diabetes mellitus or prior myocardial revascularization, and regardless of the positive history of previous MI.

The IPD meta-analysis by Kotanidis *et al*.^17^ did not find a significant effect of invasive management on the composite of all-cause mortality and MI using unadjusted Cox regression analysis with random effect in the subgroup of older female patients with NSTEACS (interaction *P*-value 0.190). However, the risk of MI was reduced by 51% in older women undergoing invasive management.^17^ These interesting findings led to further investigation on the correlation between invasive or conservative management and female sex in older NSTEACS patients in this analysis. In an unadjusted analysis, an invasive strategy did not reduce the risk of primary outcome. The prognostic role of the invasive management for the primary endpoint becomes significant in the multivariable adjusted regression model. The adjusted model accounts for the influence of age, hypertension, and diabetes mellitus, which were masking the true effect of invasive treatment on the primary endpoint in older women with NSTEACS. The After Eighty study revealed benefit from an invasive strategy in the female population, but the trend of intervention rate was lower in women compared with men. This was explained with the less significant obstructive coronary artery disease found in women compared with men (32% vs. 14%, *P* = 0.001).^23–25^

Previous studies did not find a significant benefit of performing an invasive treatment in female patients with NSTEACS.^26,27^ A pooled data analysis from the FRISC II, ICTUS, and RITA-3 (FIR) studies compared 5467 patients with NSTEACS, of whom 306 (5.60%) female aged ≥75 years, undergoing routine or selective invasive management.^28^ No significant benefit was found in women of any age categories receiving routine invasive management as compared with those allocated to a selective invasive strategy consisting of coronary angiography and revascularization driven by refractory angina, haemodynamic, or rhythmic instability despite optimal medical therapy. In particular, older women (≥75 years old) showed no significant reduction of cardiovascular death and MI (HR 0.87, 95% CI 0.57–1.33, *P* = 0.52).^28^ In our analysis, the association between invasive management and reduced risk of MI was strongly significant. No correlation was found between invasive management and cardiovascular death or all-cause mortality, although cardiovascular death was not reported as endpoint in one of the RCTs included. This is in line with previous results found in the literature.^17,29^ In the older population, mortality seems to be driven primarily by the burden of comorbidities despite the use of recommended management.^30,31^ However, there were numerically fewer cardiovascular deaths among the patients in the invasive strategy group than in the conservative strategy group (8.3% vs. 10.5%), although this difference did not reach statistical significance. In the Kaplan–Meier curve, the cumulative incidence of cardiovascular death appears to be lower in the invasive group only after 270 days. Therefore, a longer-term follow-up could have yielded different results. Nevertheless, the recently published trial, SENIOR-RITA, did not show any significant difference in cardiovascular death between the invasive and conservative groups in a combined older population of female and male patients over a median follow-up of 4.1 years.^29^

In a meta-analysis including eight trials published between 1995 and 2005 comparing invasive vs. conservative strategy in women and men with NSTEACS, invasive management reduced the risk of composite endpoint including death, MI, and rehospitalization with ACS only in females with positive cardiac biomarkers [odds ratio (OR) 0.67, 95% CI, 0.50–0.88]. No difference between treatment strategies was found in the overall female population (OR 0.81, 95% CI 0.65–1.01).^32^ In our analysis using IPD, the randomized trials included were more recent (between 2012 and 2023) and focused exclusively on older patients.^18–23^ In the subgroup with positive troponin, the risk of MI was lower in women allocated to the invasive strategy than in those allocated in the conservative strategy. It is noteworthy that the benefit of an invasive strategy was greater in women with elevated troponin on admission and without markers of a more advanced disease, such as diabetes mellitus, prior PCI, and prior CABG.

In our study, no statistically significant difference in stroke was found between the two treatment strategy groups. Nevertheless, the risk of stroke tends to be higher in older women undergoing invasive strategy. Large observational registries have shown increased risk of stroke in female patients undergoing PCI due to the risk of intra-procedural stroke during left heart catheterization.^33,34^ In our study, the intra-procedural stroke does not influence the results since there were no events within 36 h of the index procedure. However, patients allocated to the invasive strategy experienced a stroke earlier than patients in the conservative group. Further studies might help to clarify these findings.

Currently, there is limited evidence regarding interventional strategies and clinical outcomes in older patients with NSTEACS, especially women.^18–23^ The older adult population with ACS encountered in clinical practice is heterogeneous, while advanced age, frailty status, and comorbidity may impact the risk of procedural complications and clinical outcomes and influence decision-making regarding invasive management.^35,36^

### Strengths and limitations

To the best of our knowledge, this is the first study using IPD that investigates clinical adverse outcomes specifically in older women with NSTEACS undergoing either invasive or conservative management in dedicated RCTs. It included IPD from all recent trials investigating interventional management strategies that specifically enrolled older patients, better reflecting contemporary clinical practice and the heterogeneous patient population encountered today.

This study has some limitations. The design of the RCTs was open-label, which might have influenced the incidence of urgent revascularization during follow-up in patients allocated to the conservative group. Bleeding events were not evaluated as an outcome due to the different endpoint definitions adopted by the constituent trials. However, bleeding rates were relatively low and comparable between both routine invasive and initially conservative strategies within the six included trials.^17^ No significant differences were found in the post-discharge antithrombotic therapy between the two groups. Similarly, data regarding frailty status, use of radial access, drug-eluting stents, angiographic characteristics, and revascularization were not available on individual patient level for all trials. An additional limitation is the lack of comprehensive data on comorbidities in our older female cohort. Comorbidities are common in older populations and can significantly influence clinical outcomes, treatment decisions, and the risk of adverse events. While multivariable adjustments were made for cardiovascular risk factors such as age, hypertension, and diabetes mellitus, more detailed information on other comorbidities (e.g. renal disease, chronic pulmonary conditions, and heart failure) was not available, which may limit the generalizability of our findings to broader populations of older women with NSTEACS.

Furthermore, endpoints were restricted to 1 year as this was the minimum follow-up period available across all six included studies.

## Conclusion

Among older women with NSTEACS, an invasive management compared with a conservative management did not significantly reduce the risk of the composite outcome of all-cause mortality or MI. However, an invasive management did reduce risk of reinfarction and urgent revascularization at 1-year follow-up.

## Lead author biography


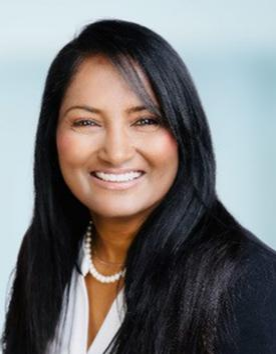


Professor Vijay Kunadian is a professor of interventional cardiology holding a personal chair at Newcastle University and an honorary academic consultant interventional cardiologist at Freeman Hospital, Newcastle upon Tyne, UK. Professor Kunadian has an international reputation in interventional cardiology and cardiovascular research, making her a sought-after and respected speaker at prominent national and international meetings. She has championed diversity in her specialty as a role model; only 5% of interventional cardiologists are female in the UK, and <1% are clinical academics/researchers and only female interventional cardiologist holding a university personal chair. She is the National Cardiovascular Specialty Group Lead, UK National Institute for Health and Care Research (NIHR) Research Delivery Network. She is the British Cardiovascular Intervention Society Research and Development Working Group lead and council member. She served as a board member of the European Association of Percutaneous Cardiovascular Interventions Executive Committee. She is a recipient of numerous awards including winner of the UK Times and the Sunday Times We Are The City Rising Star Award (Top 5 Women in Healthcare Category) for 2018. In 2022, she was nominated for the BHF Heart Hero Research Engagement Award. She was a finalist for the 2023 Northern Power Women Awards—Disruptor for Good Category. She was voted Inspirational Woman at Newcastle University in 2023. She is the UK Chief Investigator/Country Lead/National Co-ordinating investigator in numerous multicentre clinical trials. She has published >220 peer-reviewed publications in major journals such as the *New England Journal of Medicine*, *the Lancet*, *Nature Reviews*, *European Heart Journal*, *Circulation*, *Journal of the American College of Cardiology*, *Heart BMJ*, and *JAMA Cardiology*.

## Supplementary Material

oeae093_Supplementary_Data

## Data Availability

Individual participant-level data used for this report are not publicly available, because they contain protected patient health information. Requests for data access should be directed to the corresponding author via email.
