# Supplementary material for: Older women with non-ST-elevation acute coronary syndrome undergoing invasive or conservative management: an individual patient data meta-analysis
Source: Eur Heart J Open. 2024 Oct 26;4(6):oeae093. doi: 10.1093/ehjopen/oeae093 (PMC11653893; doi:10.1093/ehjopen/oeae093)
Supplement: oeae093_Supplementary_Data [file oeae093_supplementary_data.docx]

**SUPPLEMENTARY MATERIAL**

**Older women with non-ST-elevation acute coronary syndrome undergoing invasive or conservative management: an individual patient data meta-analysis**

Francesca Rubino MD, Graziella Pompei MD, Gregory B. Mills MBBS MRes, Christos P. Kotanidis MD MSc DPhil, Claudio Laudani MD MSc, Bjørn Bendz MD, Erlend S. Berg MD, David Hildick-Smith MD, Geir Hirlekar PhD, Nuccia Morici MD, Aung Myat MD, Nicolai Tegn MD^f^, Juan Sanchis Forés MD, Stefano Savonitto MD, Stefano De Servi MD, Vijay Kunadian MD

TABLE OF CONTENTS

[Supplementary Table 1. 3](#_Toc172669059)

[Supplementary Table 2 4](#_Toc172669060)

[Supplementary Table 3 6](#_Toc172669061)

[Supplementary Table 4 7](#_Toc172669062)

[Supplementary Table 5. 8](#_Toc172669063)

[Supplementary Figure 1. 9](#_Toc172669064)

[Supplementary Figure 2 10](#_Toc172669065)

[Supplementary Figure 3. 11](#_Toc172669066)

[Supplementary Figure 4 12](#_Toc172669067)

**Supplementary Table 1. Key features of the included trials**

| **Study** | **Enrolment time** | **Country** | **Population** | **Women included** | **Median time of Angiography** |
| --- | --- | --- | --- | --- | --- |
| Savonitto et al. 2012  Italian Elderly ACS | January 2008 – May 2010 | Italy | NSTEACS ≥ 75 years | 156/313 (47.9%) | 1 day |
| Tegn et al.  2016  After Eighty | December 2010 – February 2014 | Norway | NSTEACS ≥ 80 years | 232/457 (50.8%) | 1.4 day |
| Sanchis et al.  2016  MOSCA | January 2012 – March 2014 | Spain | NSTEMI ≥ 70 years | 50/106 (47.2%) | Not available |
| Hirelekar et al.  2020  80+ Study | September 2009- September 2017 | Sweden | NSTEACS ≥ 80 years | 84/186 (45.2%) | Not available |
| De Belder et al.  2021  RINCAL | May 2014 – September 2018 | United Kingdom | NSTEACS ≥ 80 years | 118/250 (47.2%) | 2 days |
| Sanchis et al.  2023  MOSCA-FRAIL | July 2017 – January 2021 | Spain | NSTEMI ≥ 70 years | 88/167 (52.7%) | Not available |
| ACS: acute coronary syndrome; MOSCA: coMOrbilidades en el Síndrome Coronario Agudo; NSTEACS: non-ST-elevation acute coronary syndrome; RINCAL: The Revascularisation or medIcal therapy iN elderly patients with aCute anginAL syndromes. | | | | | |

**Supplementary Table 2. Endpoint definitions in the included trials**

| **Trial** | **Primary composite endpoint** | **Non-fatal MI** | **Stroke** | **Urgent revascularisation** | **Bleeding** |
| --- | --- | --- | --- | --- | --- |
| Savonitto et al.; 2012  Italian Elderly ACS | All-cause mortality, reinfarction, stroke and readmission for cardiovascular causes or severe bleeding. | New cardiac symptoms with ECG evidence and elevated cardiac biomarkers > 99^th^ centile. | New focal neurological deficit with duration > 24 hours confirmed by appropriate imaging, with classification as ‘disabling’ or non-disabling. | Coronary revascularisation due to severe recurrent ischemia, or an ACS. | ‘Severe’ bleeding leading to hospital admission, including BARC ≥2 criteria. |
| Tegn et al.;  2016  After Eighty | All-cause mortality, reinfarction, urgent revascularisation and stroke | New cardiac symptoms with troponin > 99^th^ percentile. Periprocedural MI defined as rise in cardiac biomarkers beyond three times the 99^th^ centile. | New focal neurological deficit of vascular origin lasting > 24 hours. | Need for urgent revascularisation in case of refractory angina, defined as as increasing angina pectoris symptoms despite optimum medical treatment with or without ECG changes as judged by the cardiologists in the hospitals. | ‘Major’ or ‘minor’ according to TIMI criteria. |
| Sanchis et al.  2016  MOSCA | All-cause mortality, reinfarction and readmission for cardiac causes (revascularisation or heart failure) | New cardiac symptoms with troponin elevation. Periprocedural MI defined as troponin elevation beyond five times the 99^th^ centile (after PCI) or ten times the 99^th^ centile (after CABG). |  | Any revascularization after the discharge. | TIMI ≥2 criteria. |
| Hirelekar et al.  2020  80+ Study | All-cause mortality, reinfarction, urgent revascularisation,  stroke and  recurrent hospitalisation  for cardiac  reasons (heart  failure or new  onset atrial  fibrillation). | New cardiac symptoms  with troponin  > 99^th^  percentile. Periprocedural MI defined as rise in cardiac biomarkers beyond three times the 99^th^ centile. | Cerebral ischaemic events including transient ischaemic attack. | Any unplanned  revascularization after the index hospitalization. | ‘Major’ or ‘minor’ according to TIMI criteria. |
| De Belder et al.;  2021  RINCAL | All-cause mortality and  reinfarction | New cardiac symptoms with troponin > 99^th^  percentile. Periprocedural MI as per the third universal definition of  MI. | New focal  neurological deficit with  duration > 24  hours confirmed by a  neurologist and  appropriate imaging. | Any unplanned  revascularisation after the index  procedure. | BARC ≥3B criteria. |
| Sanchis et al.; 2023  MOSCA-FRAIL | Cardiac death, reinfarction and post-discharge revascularisation.  Number of days alive and out of hospital (encompassing mortality and hospitalisations). | New chest pain and troponin elevation consistent with most recent universal definition of myocardial infarction. |  | Any revascularization after the discharge. | Any bleeding requiring hospitalisation. |
| ACS: acute coronary syndrome; BARC: Bleeding Academic Research Consortium; CABG: coronary artery bypass graft; ECG: electrocardiogram; MI: myocardial infarction; TIMI: Thrombolysis in Myocardial Infarction. | | | | | |

**Supplementary Table 3. Baseline characteristics in female and male population**

| **Variables** | **Female patients**  **N=717** | **Male patients**  **N=762** | **P-value** |
| --- | --- | --- | --- |
| **Demographic data** |  |  |  |
| Age, years (IQR) | 84.0 (81.0-87.0) | 84.0 (81.0-87.0) | 0.369 |
| **Past medical history** | | | |
| Hypertension, n (%)  Missing data, (%) | 527 (73.5)  6 (0.8) | 530 (69.9)  2 (0.3) | 0.070 |
| Diabetes mellitus, n (%)  Missing data, (%) | 211 (29.4)  5 (0.7) | 212 (27.8)  2 (0.3) | 0.497 |
| Smoking status  Active smokers, (%)  Former smokers, (%)  Never smokers, (%)  Missing data, (%) | 33 (4.6)  127 (17.7)  383(53.6)  174 (24.3) | 36 (4.7)  305 (40.0)  247 (32.4)  174 (22.8) | <0.001 |
| Previous MI, (%)  Missing data, (%) | 200 (27.9)  8 (1.1) | 322 (42.3)  4 (0.5) | <0.001 |
| Previous CABG, (%)  Missing data, (%) | 54 (7.5)  7 (1.0) | 135 (17.7)  2 (0.3) | <0.001 |
| Previous PCI, (%)  Missing data, (%) | 98 (13.7)  8 (1.1) | 191 (25.1)  3 (0.4) | <0.001 |
| Previous stroke, n (%)  Missing data, (%) | 106 (14.8)  6 (0.8) | 119 (15.6)  2 (0.3) | 0.744 |
| Killip class  1  2  3  4  Missing data, (%) | 461 (64.3)  121 (16.9)  12 (1.7)  2 (0.3)  121 (16.9) | 479 (62.9)  136 (17.8)  18 (2.4)  3 (0.4)  126 (16.5) | 0.688 |
| **Medical therapy at discharge** | | | |
| Aspirin, n (%)  Missing data, (%) | 587 (81.9)  17 (2.4) | 641 (84.1)  10 (1.3) | 0.512 |
| P2Y12 inhibitors, (%)  Missing data, (%) | 582 (81.2)  15 (2.1) | 624 (81.9)  10 (1.3) | 1.000 |
| Beta blockers, n (%)  Missing data, (%) | 451 (62.9)  16 (2.2) | 456 (59.8)  8 (1.0) | 0.143 |
| ACE inhibitors, (%)  Missing data, (%) | 328 (45.7)  17 (2.4) | 369 (48.4)  11 (1.4) | 0.415 |
| Anticoagulation, (%)  Missing data, (%) | 86 (12.0)  58 (8.1) | 117 (15.4)  64 (8.4) | 0.066 |
| Statin, n (%)  Missing data, (%) | 564 (78.7)  17 (2.4) | 617 (81.0)  12 (1.6) | 0.446 |
| ACE: angiotensin converting enzyme inhibitors; CABG: coronary artery bypass graft; IQR: interquartile range; MI: myocardial infarction; n: number; PCI: percutaneous coronary artery disease. | | | |

**Supplementary Table 4. Unadjusted and adjusted Cox regression analysis for primary and secondary endpoints using fixed effect in the female population.**

|  | **HR (95%CI)** | **P-value** |
| --- | --- | --- |
| **Primary endpoint** | | |
| Invasive management |  |  |
| Univariable unadjusted model | 0.72 (0.53-0.97) | 0.030 |
| Multivariable adjusted model^*^ | 0.71 (0.53-0.96) | 0.027 |
| **All-cause death** | | |
| Invasive management |  |  |
| Univariable unadjusted model | 0.91 (0.61-1.35) | 0.635 |
| Multivariable adjusted model^*^ | 0.89 (0.60-1.33) | 0.585 |
| **Cardiovascular death** | | |
| Invasive management |  |  |
| Univariable unadjusted model | 0.80 (0.47-1.37) | 0.404 |
| Multivariable adjusted model^*^ | 0.78 (0.46-1.32) | 0.356 |
| **Myocardial infarction** | | |
| Invasive management |  |  |
| Univariable unadjusted model | 0.49 (0.33-0.74) | <0.001 |
| Multivariable adjusted model^*^ | 0.48 (0.32-0.73) | <0.001 |
| **Urgent revascularization** | | |
| Invasive management |  |  |
| Univariable unadjusted model | 0.50 (0.30-0.84) | 0.008 |
| Multivariable adjusted model^*^ | 0.50 (0.30-0.83) | 0.007 |
| **Stroke** | | |
| Invasive management |  |  |
| Univariable unadjusted model | 2.89 (0.94-8.95) | 0.066 |
| Multivariable adjusted model^*^ | 2.94 (0.94-9.15) | 0.062 |
| CI: confidence interval; HR: hazard ratio.  *Correction variables: age, hypertension and diabetes mellitus. | | |

**Supplementary Table 5. Unadjusted and adjusted Cox regression analysis for primary and secondary endpoints using random effect in the male population.**

| **Endpoints** | **Invasive**  **(N= 368)** | **Conservative**  **(N= 394)** | **Unadjusted HR (95% CI)** | **P-value** | **Adjusted HR* (95% CI)** | **P-value** |
| --- | --- | --- | --- | --- | --- | --- |
| Primary endpoint | 104 (28.3%) | 118 (29.9%) | 0.96 (0.68,1.36) | 0.810 | 0.93  (0.68-0.1.30) | 0.640 |
| All-cause death | 62 (16.8%) | 65 (16.5%) | 1.08  (0.72-1.62) | 0.720 | 0.99  (0.69-1.40) | 0.930 |
| Cardiac death^‡^ | 32/305 (10.5%) | 36/318  (11.3%) | 0.96  (0.54-1.71) | 0.900 | 0.94  (0.51-1.73) | 0.840 |
| Myocardial infarction | 55 (14.9%) | 77 (19.5%) | 0.75  (0.50-1.32) | 0.170 | 0.75  (0.50-1.12) | 0.160 |
| Urgent  revascularization^‡^ | 36/366 (9.84%) | 54/392  (13.7%) | 0.39  (0.15-1.25) | 0.110 | 0.43  (0.16-1.17) | 0.099 |
| Stroke | 10 (2.72%) | 8 (2.03%) | 0.86  (0.34-2.18) | 0.750 | 0.85  (0.33-2.16) | 0.740 |
| CI: confidence interval; HR: hazard ratio.  * Correction variables: age, hypertension and diabetes mellitus.  ^‡^ For those variables with missing outcomes data, values are reported as n/ available observations (%) | | | | | | |

**Supplementary Figure 1. Primary endpoint at 1-year follow-up**

Kaplan-Meier curves show higher cumulative incidence of primary endpoint in older women undergoing conservative management (red) than in those undergoing invasive treatment (blue).

**
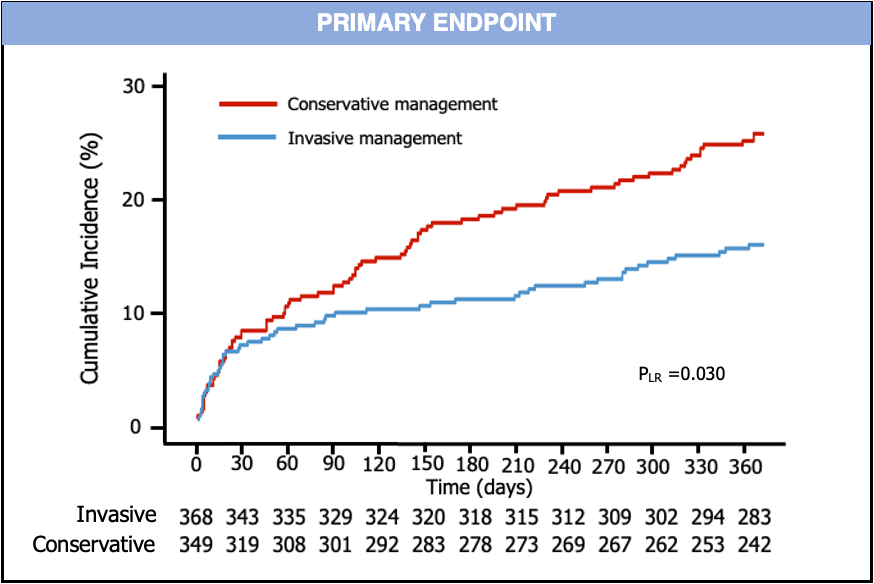
**

LR: log-rank.

**Supplementary Figure 2. Adjusted Cox regression analysis for primary and secondary endpoints using random effect in the female population.** Forest Plot shows adjusted Cox regression analysis using random effect. CI: confidence interval; HR: hazard ratio.

**
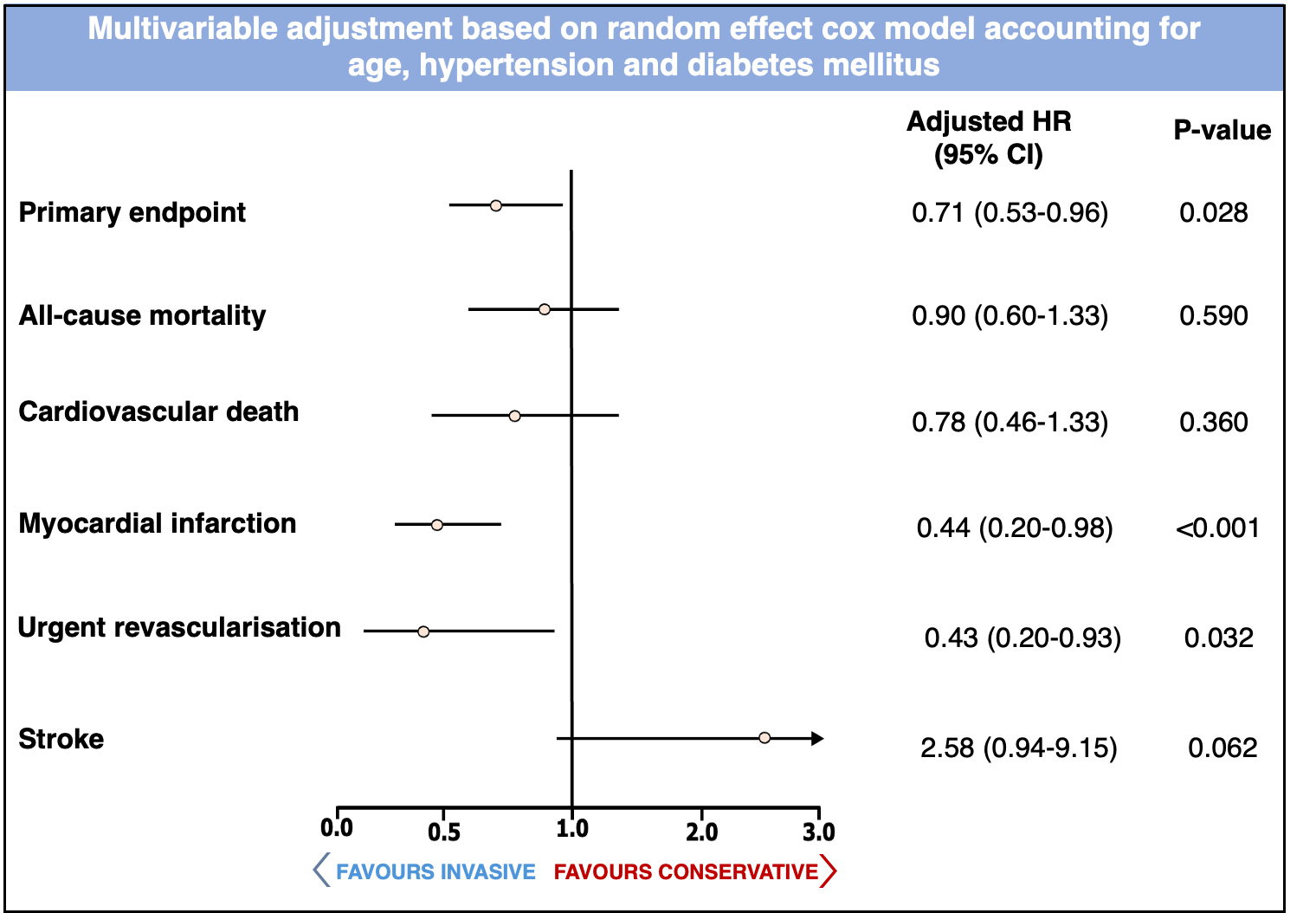
**

**Supplementary Figure 3. All-cause mortality and cardiovascular death incidence in older women undergoing invasive or conservative management.**

Kaplan-Meier Curves show no significant difference between older women undergoing conservative management (red) than in those undergoing invasive treatment (blue).

**
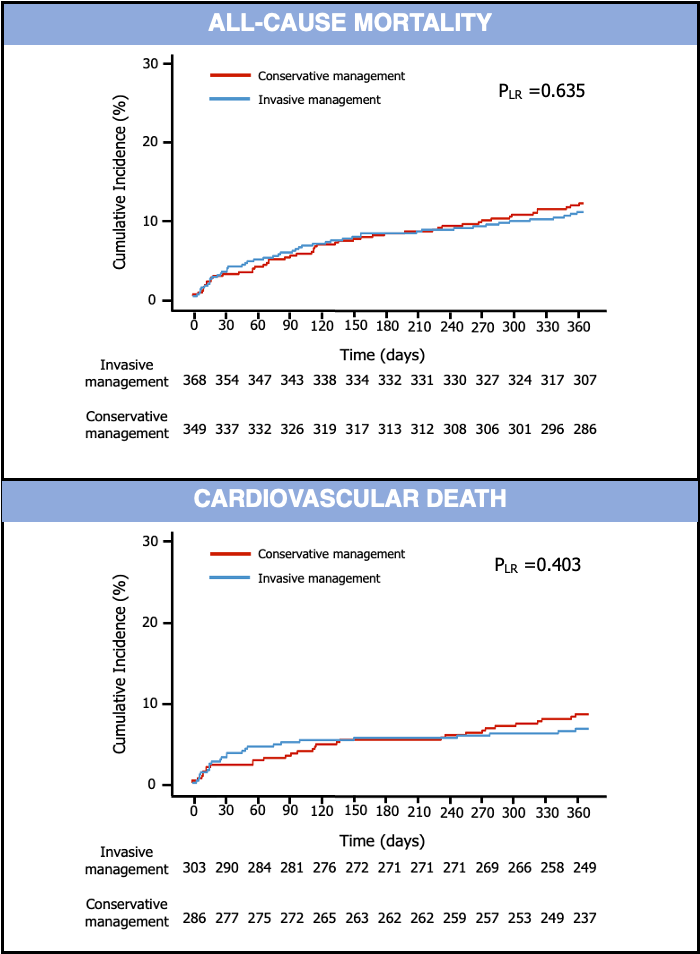
**

LR: log-rank.

**Supplementary Figure 4. Stroke cumulative incidence in older women undergoing invasive or conservative management.**

Kaplan-Meier Curves show no significant difference between older women undergoing conservative management (red) than in those undergoing invasive treatment (blue).

**
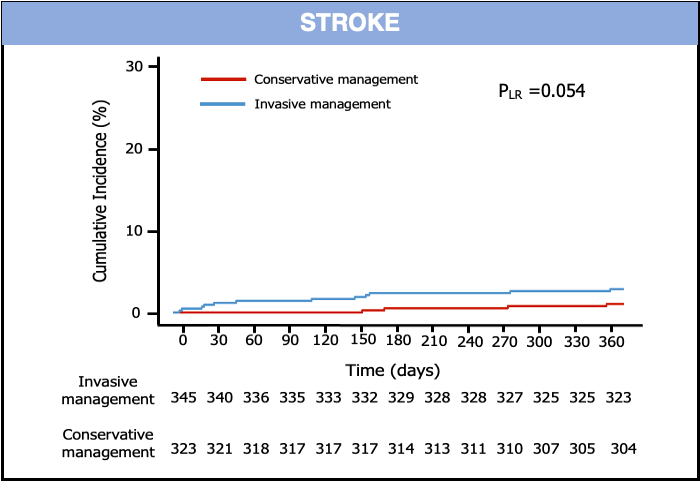
**

LR: log-rank.
